# Investigating bias in squared regression structure coefficients

**DOI:** 10.3389/fpsyg.2015.00949

**Published:** 2015-07-08

**Authors:** Kim F. Nimon, Linda R. Zientek, Bruce Thompson

**Affiliations:** ^1^Department of Human Resource Development, University of Texas at TylerTyler, TX, USA; ^2^Department of Mathematics & Statistics, Sam Houston State UniversityHuntsville, TX, USA; ^3^Department of Educational Psychology, Texas A&M University, College StationTX, USA

**Keywords:** structure coefficients, beta weights, multiple linear regression, general linear model

## Abstract

The importance of structure coefficients and analogs of regression weights for analysis within the general linear model (GLM) has been well-documented. The purpose of this study was to investigate bias in squared structure coefficients in the context of multiple regression and to determine if a formula that had been shown to correct for bias in squared Pearson correlation coefficients and coefficients of determination could be used to correct for bias in squared regression structure coefficients. Using data from a Monte Carlo simulation, this study found that squared regression structure coefficients corrected with Pratt's formula produced less biased estimates and might be more accurate and stable estimates of population squared regression structure coefficients than estimates with no such corrections. While our findings are in line with prior literature that identified multicollinearity as a predictor of bias in squared regression structure coefficients but not coefficients of determination, the findings from this study are unique in that the level of predictive power, number of predictors, and sample size were also observed to contribute bias in squared regression structure coefficients.

## Investigating bias in regression squared structure coefficients

Empirical reviews of published analytic practices show that multiple regression has been a widely used statistical method within the social sciences (cf. Willson, 1980; Goodwin and Goodwin, 1985; Elmore and Woehlke, 1988; Kieffer et al., 2001; Leach and Henson, 2007). In such studies, it is not unusual for predictors to be correlated. Increases in multicollinearity are problematic because multicollinearity can inflate variances of regression coefficients, and can complicate the ability to identify the importance of predictor variables (Stevens, 2002).

In the presence of correlated predictors, Courville and Thompson (2001) advised researchers to use structure coefficients or correlation coefficients in addition to β weights when interpreting the results of multiple regression. While β weights indicate the predicted change in the standardized dependent variable for every unit change in a given standardized predictor variable, holding all other predictors constant, squared structure coefficients indicate how much of the regression effect can be attributed to a given predictor. It is possible for a predictor to have a low β weight and a high structure coefficient (indicative of multicollinearity) or a high β weight and a low structure coefficient (indicative of suppression). Without considering both sets of coefficients, researchers may incorrectly interpret a predictor as making little to no contribution to the regression effect because its contribution is being masked due to multicollinearity as well as miss the presence of suppression. As such, both sets of coefficients are needed to help researchers interpret regression results in the presence of multicollinearity. Additionally, the coefficients answer different research questions. As noted by Nathans et al. (2012), β weights can be used the answer the question, “What is the contribution of each independent variable to the regression equation, holding all other independent variables constant? (p. 3) and squared structure coefficients can be used the answer the question, “How much variance in the predicted scores for the dependent variable ($\hat{\text{y}}$) can be attributed to each independent variable when variance is allowed to be shared between independent variables?” (p. 7).

In the present study, we investigated the bias of squared regression structure coefficients and determined if a formula, that has been used to correct for bias in coefficients of determination and Pearson *r*^2^, could be used to correct for bias in squared regression structure coefficients. Squared regression structure coefficients with less bias will be more true to the population parameters and more accurately describe how much of the regression effect can be attributed to a given predictor. In the remainder of this section, we review the general linear model (GLM) as a rubric for regression interpretation followed by the squared regression structure coefficient, squared multiple correlation coefficient, Pearson *r*^2^, and sample sizes in published literature before presenting the purpose of the study.

## General linear model (GLM) as a rubric for regression interpretation

Multiple regression analyses are part of the GLM. Furthermore, all analytic methods that are part of the GLM are correlational and have the capability of producing variance-accounted-for effect sizes such as *R*^2^, η^2^, ω^2^, which are analogs to *r*^2^ (see Thompson, 2000, 2006; Zientek and Thompson, 2009). As Graham (2008) further explained,

The vast majority of parametric statistical procedures in common use are part of (a single analytic family called) the GLM, including the *t*-test, analysis of variance (ANOVA), multiple regression, descriptive discriminant analysis (DDA), multivariate analysis of variance (MANOVA), canonical correlation analysis (CCA), and structural equation modeling (SEM). Moreover, these procedures are *hierarchical* (italics added), in that some procedures are special cases of others. (p. 485).

The hierarchical structure of the GLM has been demonstrated by the work of several researchers. First, Cohen (1968) showed that all univariate parametric analyses such as *t-*tests, ANOVAs, and Pearson *r* are subsumed as special cases of multiple regression analysis. Next, Knapp (1978) showed that all of the common univariate *and* multivariate analyses conducted in research are special cases of canonical correlation analysis. Finally, Bagozzi et al. (1981) and later Graham (2008) showed that SEM can be categorized as an even more general case of the GLM (see Fan, 1997 for more detail).

The importance of interpreting structure coefficients and analogs of regression weights for statistical analyses within the GLM permeates the literature. For example, within the GLM for the exploratory factor analysis case, Gorsuch (1983) argued that the interpretation of factors is contingent on the factor structure. Graham et al. (2003) made a similar argument with respect to the importance of interpreting both factor pattern coefficients–the analogs of regression β weights–and structure coefficients in confirmatory factor analysis.

Kerlinger and Pedhazur (1973) noted that the weights that are analogs to regression β weights emerge as a weak link in the canonical correlation chain. According to Meredith (1964), when variables are moderately intercorrelated, there is the possibility that interpretations of canonical variables will be nearly nil by inspection of regression weights (function coefficients). Thus, Thompson and Borrello (1985; also see Dunlap and Landis, 1998) argued that

If structure coefficients rather than function coefficients should be interpreted in the canonical case, logic suggests that perhaps structure coefficients should be interpreted in the regression case, since the two methods are actually identical. (p. 205).

Or, perhaps more appropriately, *both* β weights and structure coefficients should be interpreted whenever regression predictor variables are correlated with each other. A review of relative importance indices further indicates that β weights and validity coefficients (zero-order correlations between given predictor variables and the dependent variable) or structure coefficients are indices that have been interpreted to determine relative importance for multiple regression results (see Johnson and Lebreton, 2004).

### Structure coefficients *r*_*x*$\hat{\text{y}}$_

Regression structure coefficients *r*_*x*$\hat{\text{y}}$_ are the bivariate correlation coefficients between given predictor variables and the latent predicted outcome variable (i.e., $\hat{Y}$). With a few simple commands, regression structure coefficients can be included in statistical output (Kraha et al., 2012). In addition, regression structure coefficients can be calculated by dividing the validity coefficient for a predictor (i.e., bivariate correlation between a predictor variable *X* and the dependent variable *Y*) by the multiple correlation coefficient: $r_{x\hat{y}}=\;\frac{r_{xy}}{R_{y\hat{y}}}$. Because regression β weights are the multiplicative weights applied to the standardized predictor variables to compute scores on the latent predicted outcome variable, simultaneously interpreting structure coefficients or validity coefficients along with β weights allows researchers to view different dynamics within the data.

Because, both the squared multiple correlation coefficient (*R*^2^_*y*$\hat{\text{y}}$_) and the squared validity coefficient (*r*^2^_*xy*_) are biased (see Yin and Fan, 2001; Skidmore and Thompson, 2011), logically, *r*^2^_*x*$\hat{\text{y}}$_ is biased. We located only one study that conducted a Monte Carlo study that included regression structure coefficients. Jiang and Smith (2002) determined that *r*_*x*$\hat{\text{y}}$_ increased as a function of multicollinearity, was relatively stable across multiple sample sizes, and increased when a strong predictor was excluded from the model. Because *R*^2^_*y*$\hat{\text{y}}$_ and *r*^2^_*xy*_ are terms in the *r*^2^_*x*$\hat{\text{y}}$_ formula $\left(r_{x\hat{y}}^{2}=\frac{r_{xy}^{2}}{R_{y\hat{y}}^{2}}\right)$ and both have been identified as being biased, a review of those statistics is warranted.

### Squared multiple correlation coefficient *R*^2^_*y*$\hat{\text{y}}$_

The squared multiple correlation coefficient *R*^2^_*y*$\hat{\text{y}}$_ has been one of the most reported effect sizes, possibly because of the pervasiveness of multiple regression analyses in social science research and the fact that *R*^2^_*y*$\hat{\text{y}}$_ is routinely and automatically produced in statistical software output (Kirk, 1996; Alhija and Levy, 2009). However, *R*^2^_*y*$\hat{\text{y}}$_ tends to be positively biased because the assumption “that the values of the independent variables are known constants and are fixed by the researcher before the experiment” is usually not met (see Yin and Fan, 2001, p. 206). In order to shrink *R*^2^_*y*$\hat{\text{y}}$_, which is the denominator of *r*^2^_*x*$\hat{\text{y}}$_, a number of correction formulas have been developed. By the late 1990s, the field had not yet decided on the best correction formula; thus, several researchers began a quest to identify the formula that created the smallest amount of bias under various conditions. The research produced inconsistent results about the best correction formula to apply, possibly because of methodological issues, such as a given simulation including a limited number of formulas investigated or using real data instead of simulated data (see Raju et al., 1999; Yin and Fan, 2001). For instance, results from Raju et al. (1999) suggested that the Ezekiel (reported as *Adjusted R Square* in standard SPSS output), Smith, and Wherry formulas were good for estimating the population squared multiple correlation coefficient (ρ^2^_*y*$\hat{\text{y}}$_). However, Raju et al. acknowledged that the use of one dataset limited their ability to take into consideration various number of predictor variables and population effect sizes. In a review of correction formulas contained in published studies, Leach and Henson (2007) showed that the Ezekiel correction was the most conservative and Claudy-3 was the least conservative correction for sampling error. In a Monte Carlo study, Yin and Fan (2001) investigated bias for six formula corrections for *R*^2^_*y*$\hat{\text{y}}$_ and found that the Pratt formula (Cureton, personal communication, October 20, 1964; as cited in Claudy, 1978, p. 597) was the best performer as an unbiased estimator for ρ^2^_*y*$\hat{\text{y}}$_ under three multicollinearity and population conditions and five *N*/*p* conditions and the Olkin and Pratt formula was the second best performer under those conditions. As indicated in Equation (1), Pratt's formula adjusts *R*^2^_*y*$\hat{\text{y}}$_ based on the sample size (*N*) and number of predictors (*p*) in a particular regression model:

(1) $$\rho_{y\hat{y}}^{2}=1-\frac{\left(N-3\right)(1-R^{2})}{N-p-1}[1+\frac{2\left(1-R^{2}\right)}{N-p-2.3}]$$

Yin and Fan's (2001) results indicated that the Pratt formula generated the smallest amount of bias for estimating ρ^2^_*y*$\hat{\text{y}}$_, particularly for relatively small ratios of *N/p*, and the Claudy formula-3 generated the largest amount of bias. Furthermore, they determined that for all of the correction formulas they investigated, when the ratio *N/p* was large, or around 100, almost all of the six correction formulas were unbiased. However, their results suggest that when *N* is around 60 and with 2 predictor variables, the Pratt and Claudy-3 formula might be the best unbiased estimator ρ^2^_*y*$\hat{\text{y}}$_; and when *N* is around 100 with 2 predictor variables, the Wherry-2 was the best unbiased estimator for ρ^2^_*y*$\hat{\text{y}}$_. Confirmation that choosing the most appropriate correction formula can be complicated is evidenced by the fact that three different correction formulas yield the smallest amount of bias when the sample size is 200 and there are 2, 4, or 8 predictor variables (i.e., Smith and Wherry-1, Claudy-3, and Wherry-2, respectively). Yin and Fan, therefore, recommended that researchers examine the results published in their Table 3 to determine the formula to use under various sample sizes and numbers of predictors. Particular attention should be given to the formulas used in statistical software because the *Adjusted R Square* reported in standard SPSS output sometimes has been *correctly* attributed to Ezekiel, and sometimes *mistakenly* credited to Wherry (cf. Leach and Henson, 2007).

As noted by Leach and Henson (2007), it would be logical that the generic factors that influence sampling error would influence the shrinkage of *R*^2^_*y*$\hat{\text{y}}$_. Sampling error decreases as (a) sample size increases, (b) the number of predictor variables decreases, and (c) population effect sizes increase (Thompson, 2006). Results from Raju et al. (1999) revealed that as sample size increases, the bias for the correction formulas for ρ^2^_*y*$\hat{\text{y}}$_ tend to decrease. In the study by Yin and Fan (2001), sample size was the most important factor that contributed to the variance of bias, although the amount of contributions of all of the factors was small.

### Squared validity coefficient *r*^2^_*xy*_

The squared validity coefficient is the Pearson *r*^2^ (herein referred to as *r*^2^) between the dependent variable Y and a given predictor variable X (*r*^2^_*xy*_). When there is one predictor variable in the model, *r*^2^_*xy*_ and *R*^2^ are equivalent. Researchers typically do not apply a correction formula to *r*^2^ even though sampling error certainly affects these estimates too. Wang and Thompson (2007) examined the bias of *r*^2^ and sought to determine under a variety of conditions the best formula for minimizing the bias of *r*^2^. They investigated five correction formulas for *R*^2^_*y*$\hat{\text{y}}$_(i.e., Claudy, Ezekiel, Olkin-Pratt, Pratt, and Smith) and applied those to *r*^2^. They found that when that when the sample sizes were small and the population effect sizes were small, *r*^2^ was biased. However, while all of the correction formulas except Claudy (1978) seemed to reasonably control bias for *r*^2^ for a variety of conditions, the Ezekiel (1929) and Smith (as cited in Ezekiel, 1929, p. 100) correction formulas appeared to be the most suitable for controlling the exhibited bias. Skidmore and Thompson (2011) built on the Wang and Thompson (2007) study by investigating absolute bias and including another correction not including in their study (i.e., Olkin and Pratt, 1958). They found that the best correction formula for *r*^2^ was the Pratt formula but that the Olkin–Pratt Extended was a viable option and the Ezekiel formula was a reasonable option. Shieh (2010) also found that the Olkin and Pratt (1958) formula resulted in a minimal amount of bias for Pearson *r*.

### Sample sizes in published research

Because sample size is an important feature of research studies, it is important to know the sample sizes typically published in research. Reviews of research have indicated that sample sizes across various content areas with the majority containing fewer than 200 participants. The review of sample sizes published in core psychological research across four journals found no statistically significant differences in the median sample size in 1955 and the sample sizes of studies published in 1977, 1995, and 2006. In 1955, they found the median of 448 sample sizes in four reviewed journals was 59.95 with a mean of 180.49 (*SD* = 193.86). In 2006, they found the median of 690 sample size was 40 with the mean sample size of 195.78 (*SD* = 680.02). The third quartile of data was 131.30 for 1955 and 136 for 2006. Thus, the largest range occurred between the top 25% of the data. In education and counseling psychology, Kieffer et al. (2001) examined sample sizes of quantitative research studies that were published over a 10-year timeframe. In their review of articles, median samples sizes for each of the 10 years in the *American Educational Research Journal* ranged from 43 to 169 and median samples sizes in the *Journal of Counseling Psychology* ranged from 76 to 139.

## Purpose of the present study

Various statistics can be interpreted when a regression effect size is deemed noteworthy, including dominance statistics and relative weights. However, as regression β weights are readily available in statistical software output, applied researchers are advised to interpret β weights alongside structure coefficients in the presence of correlated predictors (Courville and Thompson, 2001). Despite realization that *R*^2^_*y*$\hat{\text{y}}$_ and *r*^2^ are positively biased, the discussion of bias has not typically included structure coefficients. If each term in the structure coefficient formula is biased, we hypothesize that structure coefficients are also biased. Even though Jiang and Smith (2002) examined *r*_*x*$\hat{\text{y}}$_ in their Monte Carlo study, they did not seek to find a correction formula for *r*^2^_*x*$\hat{\text{y}}$_.

The purpose of the present study was to investigate bias in *r*^2^_*x*$\hat{\text{y}}$_ across a number of study conditions to determine if there was sufficient bias to warrant correction, and, if so, to determine if a formula (i.e., Pratt's) that had been shown to correct for bias in *r*^2^ and *R*^2^_*y*$\hat{\text{y}}$_ could be used to correct for bias in *r*^2^_*x*$\hat{\text{y}}$_. Investigating bias under additional conditions than have been previously considered (cf., Jiang and Smith, 2002) and analyzing the effects of applying relevant correction formula will advance researchers' abilities to interpret MR results. The sample sizes were chosen that were reflective of articles published in education, psychology, and counseling (Kieffer et al., 2001; Marszalek et al., 2011).

## Method

We conducted a Monte Carlo simulation to investigate the bias of *r*^2^_*x*$\hat{\text{y}}$_ under the same study conditions in which Yin and Fan (2001) investigated the bias of corrected *R*^2^_*y*$\hat{\text{y}}$_. These study conditions included three population squared multiple correlation coefficients (ρ^2^_*y*$\hat{\text{y}}$_ = 0.20, 0.50, 80), three levels of multicollinearity among the predictors in the population (ρ ^2^_*xx*_ = 0.10, 0.30, 50), five sample sizes (*n* = 20, 40, 60, 100, 200), and three levels of predictor set size (*k* = 2, 4, 8). As in Yin and Fan (2001), we choose the “correlation coefficients between the dependent and independent variables to yield the desired squared population multiple correlation coefficient” (p. 213) and modeled the correlations among the independent variables to be the same (e.g., ρ_*x*1*x*2_ = ρ_*x*1*x*3_). As can be seen in Table 1, the population squared validity coefficients (ρ ^2^_*xy*_) varied from 0.11 to 0.60 and the population squared structure coefficients (ρ ^2^_*x*$\hat{\text{y}}$_) varied from 0.21 to 0.75 as a function of ρ^2^_*y*$\hat{\text{y}}$_, ρ_*xx*_, and *k*.

**Table 1:** **Population parameter study conditions**.

***k***	**ρ^2^_*y*$\hat{\text{y}}$_**	**ρ_*xx*_**	**ρ^2^_*xy*_**	**ρ^2^_*x*$\hat{\text{y}}$_**
2	0.2	0.1	0.11	0.55
2	0.5	0.1	0.28	0.55
2	0.8	0.1	0.44	0.55
2	0.2	0.3	0.13	0.65
2	0.5	0.3	0.33	0.65
2	0.8	0.3	0.52	0.65
2	0.2	0.5	0.15	0.75
2	0.5	0.5	0.38	0.75
2	0.8	0.5	0.60	0.75
4	0.2	0.1	0.07	0.33
4	0.5	0.1	0.16	0.33
4	0.8	0.1	0.26	0.33
4	0.2	0.3	0.10	0.48
4	0.5	0.3	0.24	0.48
4	0.8	0.3	0.38	0.48
4	0.2	0.5	0.13	0.63
4	0.5	0.5	0.31	0.63
4	0.8	0.5	0.50	0.63
8	0.2	0.1	0.04	0.21
8	0.5	0.1	0.11	0.21
8	0.8	0.1	0.17	0.21
8	0.2	0.3	0.08	0.39
8	0.5	0.3	0.19	0.39
8	0.8	0.3	0.31	0.39
8	0.2	0.5	0.11	0.56
8	0.5	0.5	0.28	0.56
8	0.8	0.5	0.45	0.56

*k, number of predictors; ρ^2^_*y*$\hat{\text{y}}$_, population squared multiple correlation coefficient; ρ_*xx*_, population correlation coefficient between predictors; ρ^2^_*xy*_, population squared validity coefficient; ρ^2^_*x*$\hat{\text{y}}$_, population squared structure coefficient*.

In total, 27 population inter-correlation matrices were derived based on the study parameters. These matrices served as the input parameters to the mvrnorm function (Venables and Ripley, 2002) in R (R Development Core Team, 2015), which was used to generate the population data for a given simulation design cell. For each cell, 1 million cases were simulated. To confirm that the code correctly created the population data, we compared the covariance matrix from each set of population data to its corresponding input covariance matrix and determined that the code was correct.

To sample from the populations, we next employed the sample function in R and the standard simulation practices outlined in Taylor et al. (2006) such that “cases were drawn without replacement within a sample but with replacement across samples” (p. 233). We drew 5000 samples under each simulation design condition to minimize the standard error of simulation.

In each of the 675,000 (3 × 3 × 5 × 3 × 5000) samples, bias was computed by subtracting known population parameters from sample estimates. Positive bias values reflect coefficients that overestimated true population parameters, while negative bias values reflect coefficients that underestimated true population parameters. We used Pratt's formula to compute corrected *R*^2^_*y*$\hat{\text{y}}$_s and *r*^2^_*xy*_s which were then used to compute corrected *r*^2^_*x*$\hat{\text{y}}$_s because Pratt's formula was shown to yield less bias in prior studies for *R*^2^_*y*$\hat{\text{y}}$_ and *r*^2^. We followed Shieh's (2008) recommendation and set negative corrected *r*^2^_*xy*_s and *R*^2^_*y*$\hat{\text{y}}$_s to zero. Corrected *r*^2^_*x*$\hat{\text{y}}$_s were set to zero for all instances when corrected *r*^2^_*xy*_s and *R*^2^_*y*$\hat{\text{y}}$_s were zero. Corrected *r*^2^_*x*$\hat{\text{y}}$_s were set to one in cases where corrected *r*^2^_*xy*_s were greater than corrected *R*^2^_*y*$\hat{\text{y}}$_s.

A multi-way ANOVA was performed on the uncorrected and corrected *r*^2^_*x*$\hat{\text{y}}$_, *R*^2^_*y*$\hat{\text{y}}$_, and *r*^2^_*xy*_ to determine the effect of the study conditions and their interactions. We used ANOVA η ^2^ values to partition the total sums of squares into non-overlapping components (cf. Wang and Thompson, 2007) and ANOVA estimated marginal means to plot bias as a function of the study parameters (cf. Skidmore and Thompson, 2011). We also computed the percentage of cells where the average bias was within the bounds of ± 0.01 in keeping with Yin and Fan (2001).

## Results

The mean bias (and *SD*) for the uncorrected *r*^2^_*x*$\hat{\text{y}}$_, *R*^2^_*y*$\hat{\text{y}}$_, and *r*^2^_*xy*_ were −0.03 (0.08), 0.04 (0.11), and 0.01 (0.06), respectively (see Table 2). The corrected versions of *r*^2^_*x*$\hat{\text{y}}$_, *R*^2^_*y*$\hat{\text{y}}$_, and *r*^2^_*xy*_ resulted in a lower set of mean bias across the study conditions: −0.010, 0.001, and 0.002, respectively. While the mean bias for the uncorrected statistics do not appear to be substantial, analyses of bias as a function of study parameters revealed cases where the amount of bias was sufficiently large enough (>|0.01|) to warrant misleading conclusions regarding the percentage of the regression effect that should validly be attributed to a predictor in the population (cf., Yin and Fan, 2001).

**Table 2:** **Statistics for bias within the 135 (3 × 3 × 3 × 5) simulation conditions**.

**Statistic/source**	**Uncorrected**			**Corrected**		
	***r*^2^_*x*$\hat{\text{y}}$_**	***R*^2^_*y*$\hat{\text{y}}$_**	***r*^2^_*xy*_**	***r*^2^_*x*$\hat{\text{y}}$_**	***R*^2^_*y*$\hat{\text{y}}$_**	***r*^2^_*xy*_**
*M*	−0.03	0.04	0.01	−0.010	0.001	0.002
*SD*	0.08	0.11	0.06	0.106	0.103	0.066
η ^2^ **VALUES FOR SIMULATION DESIGN FACTORS FOR BIAS**						
*n*	4.10%	1.56%	1.38%	2.35%	0.06%	0.09%
ρ^2^_*y*$\hat{\text{y}}$_	8.94%	5.75%	0.54%	1.72%	0.05%	0.03%
ρ^2^_*xx*_	4.85%	0.01%	0.07%	0.27%	0.01%	0.00%
*k*	3.22%	6.89%	0.12%	0.06%	0.01%	0.02%
*n:*ρ^2^_*y*$\hat{\text{y}}$_	2.27%	3.37%	0.35%	3.98%	0.17%	0.10%
*n:*ρ^2^_*xx*_	2.03%	0.00%	0.08%	0.64%	0.00%	0.02%
ρ^2^_*y*$\hat{\text{y}}$_ : ρ^2^_*xx*_	3.90%	0.01%	0.03%	1.11%	0.01%	0.07%
*n:k*	0.64%	4.16%	0.07%	0.03%	0.02%	0.02%
ρ^2^_*y*$\hat{\text{y}}$_ : *k*	2.16%	1.49%	0.04%	0.04%	0.01%	0.01%
ρ^2^_*xx*_ : *k*	2.50%	0.01%	0.09%	0.36%	0.01%	0.06%
*n:*ρ^2^_*y*$\hat{\text{y}}$_ : ρ^2^_*xx*_	0.63%	0.00%	0.01%	0.77%	0.00%	0.01%
*n:*ρ^2^_*y*$\hat{\text{y}}$_ : *k*	0.34%	0.88%	0.01%	0.05%	0.04%	0.00%
*n:*ρ^2^_*xx*_ : *k*	0.36%	0.00%	0.01%	0.12%	0.00%	0.01%
ρ^2^_*y*$\hat{\text{y}}$_ : ρ^2^_*xx*_ : *k*	1.17%	0.03%	0.03%	0.37%	0.02%	0.03%
*n:*ρ^2^_*y*$\hat{\text{y}}$_ : ρ^2^_*xx*_ : *k*	0.06%	0.01%	0.01%	0.10%	0.01%	0.00%
Total	37.17%	24.17%	2.84%	11.97%	0.42%	0.47%

**r*^2^_*x*$\hat{\text{y}}$_, sample squared structure coefficient; *R*^2^_*y*$\hat{\text{y}}$_, sample squared multiple correlation coefficient; *r*^2^_*xy*_, sample squared validity coefficient; n, sample size; ρ^2^_*y*$\hat{\text{y}}$_, population squared multiple correlation coefficient; ρ*_*xx*_*, population correlation coefficient between predictors; k, number of predictors*.

**Figure 1:**
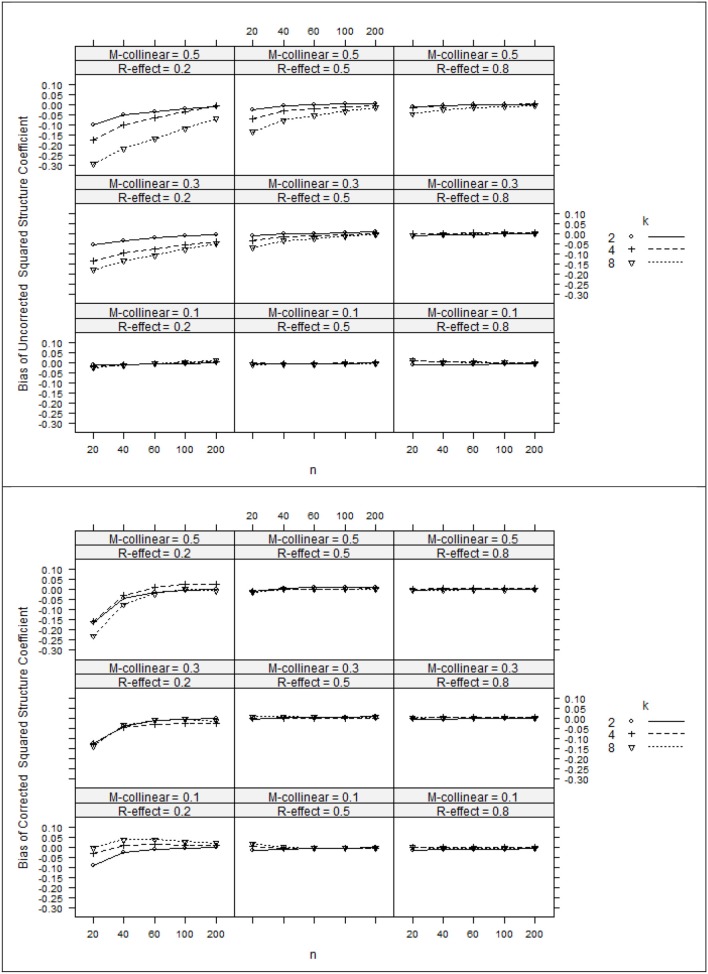
**Bias of uncorrected squared structure coefficients (top panel) and corrected squared structure coefficients (bottom panel). ***k***, number of predictors; ***n***, sample size**.

**Figure 2:**
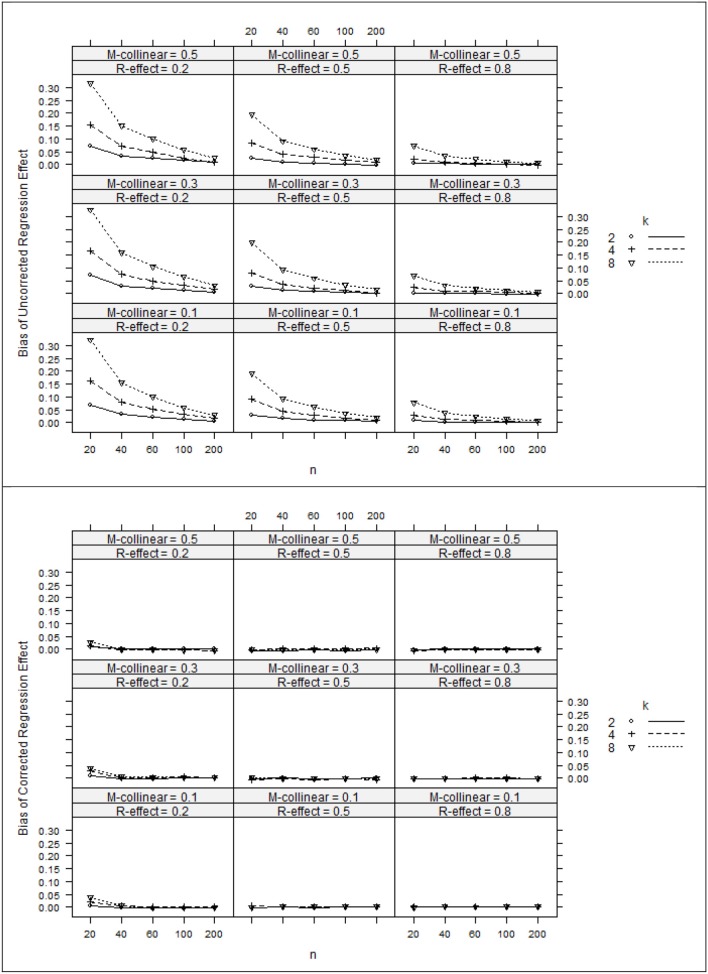
**Bias of regression effects (top panel) and corrected regression effects (bottom panel). ***k***, number of predictors; ***n***, sample size**.

**Figure 3:**
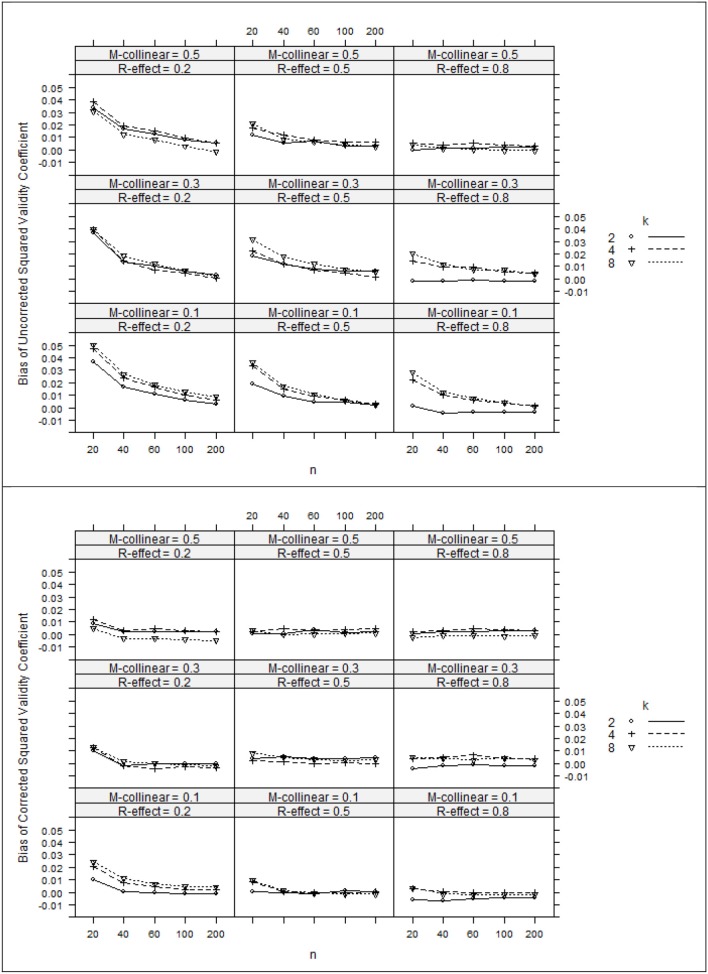
**Bias of squared validity coefficients (top panel) and corrected squared validity coefficients (bottom panel). ***k***, number of predictors; ***n***, sample size**.

The ANOVA η ^2^ values (see Table 2) suggest that the negative bias in *r*^2^_*x*$\hat{\text{y}}$_ appeared to be mostly a function of the study's main effects as well as number of interaction effects including *n:*ρ^2^_*y*$\hat{\text{y}}$_, ρ^2^_*y*$\hat{\text{y}}$_*:*ρ^2^_*xx*_, ρ^2^_*y*$\hat{\text{y}}$_*:k*, and ρ ^2^_*xx*_*:k*. However, the ANOVA estimated marginal means tell somewhat of a different story (see Figure 1). When ρ^2^_*y*$\hat{\text{y}}$_ = 0.80, bias was minimal as long as the level of multicollinearity = 0.30 or 0.10. When ρ^2^_*y*$\hat{\text{y}}$_ = 0.50 and ρ^2^_*y*$\hat{\text{y}}$_ = 0.20, bias was minimal as long as the level of multicollinearity = 0.10. In other instances, bias appeared to be a factor of *k* and *n*, with the greatest impact being seen in the case when ρ^2^_*y*$\hat{\text{y}}$_ = 0.20 and ρ^2^_*xx*_ = 0.50. The role that *k* and *n* plays in the bias of *r*^2^_*x*$\hat{\text{y}}$_ appears to stem from related bias in *R*^2^_*y*$\hat{\text{y}}$_, where the interaction between *k* and *n* appears to be a function of ρ^2^_*y*$\hat{\text{y}}$_ (see Figure 2). It would also appear that the role that sample size play in the bias of *r*^2^_*xy*_ contributes to the bias of *r*^2^_*x*$\hat{\text{y}}$_ (see Figure 3).

After applying Pratt's formula to *r*^2^_*x*$\hat{\text{y}}$_, *R*^2^_*y*$\hat{\text{y}}$_, and *r*^2^_*xy*_, we see that with few exceptions, study parameters played little role in identifying the remaining bias in the corrected estimates (see Table 2, Figures 1–3). The notable exception is when ρ^2^_*y*$\hat{\text{y}}$_ = 0.20 and *n* = 20. In this case, positive bias in ρ^2^_*y*$\hat{\text{y}}$_ generally increased as *k* increased. For *r*^2^_*x*$\hat{\text{y}}$_, whether *k* generated positive or negative bias was a function of ρ ^2^_*xx*_ (see Figures 1, 2).

The impact of the study conditions can also be seen in Table 3 that outlines the proportions of cell conditions in which unbiased estimated where observed across the study's main effect. Across *r*^2^_*x*$\hat{\text{y}}$_, *R*^2^_*y*$\hat{\text{y}}$_, and *r*^2^_*xy*_, the proportion of cell conditions with unbiased estimates were generally higher for corrected estimates across all levels of the study's main effects with the exception of *r*^2^_*x*$\hat{\text{y}}$_ when ρ ^2^_*xx*_ = 0.10. In addition, it would appear that further work is necessary to produce accurate and stable estimates of ρ^2^_*x*$\hat{\text{y}}$_, particularly when ρ^2^_*y*$\hat{\text{y}}$_ and *n* are small.

**Table 3:** **Proportions of cell conditions in which unbiased estimates observed across main effects**.

**Main effect**	**Uncorrected**			**Corrected**		
	***r*^2^_*x*$\hat{\text{y}}$_**	***R*^2^_*y*$\hat{\text{y}}$_**	***r*^2^_*xy*_**	***r*^2^_*x*$\hat{\text{y}}$_**	***R*^2^_*y*$\hat{\text{y}}$_**	***r*^2^_*xy*_**
***ρ*^2^_*y*$\hat{\text{y}}$_**						
0.20	0.27	0.09	0.40	0.33	0.84	0.82
0.50	0.60	0.24	0.62	0.91	1.00	1.00
0.80	0.87	0.62	0.84	0.96	1.00	1.00
**ρ^2^_*xx*_**						
0.10	0.82	0.29	0.53	0.71	0.96	0.91
0.30	0.53	0.31	0.60	0.76	0.96	0.93
0.50	0.38	0.36	0.73	0.71	0.93	0.98
***n***						
20	0.26	0.11	0.19	0.52	0.74	0.74
40	0.56	0.19	0.33	0.67	1.00	0.96
60	0.63	0.33	0.67	0.74	1.00	1.00
100	0.67	0.33	0.93	0.85	1.00	1.00
200	0.78	0.63	1.00	0.85	1.00	1.00
***k***						
2	0.73	0.62	0.71	0.76	0.71	0.96
4	0.60	0.27	0.60	0.69	0.60	0.93
8	0.40	0.07	0.56	0.73	0.56	0.93

**r*^2^_*x*$\hat{\text{y}}$_ = sample squared structure coefficient. *R*^2^_*y*$\hat{\text{y}}$_ = sample squared multiple correlation coefficient. *r*^2^_*xy*_ = sample squared validity coefficient. ρ ^2^_*y*$\hat{\text{y}}$_ = population squared multiple correlation coefficient. ρ_*xx*_ = population correlation coefficient between predictors. n = sample size. k = number of predictors*.

## Discussion

Using data from a Monte Carlo simulation, we found that *r*^2^_*x*$\hat{\text{y}}$_ computed from *R*^2^_*y*$\hat{\text{y}}$_ and *r*^2^_*xy*_ corrected with Pratt's formula produced less biased estimates and more stable estimates of ρ ^2^_*y*$\hat{\text{y}}$_ than estimates with no such corrections. The findings from this study are in line with prior literature that identified multicollinearity as a predictor of bias in *r*_*x*$\hat{\text{y}}$_ but not *R*^2^_*y*$\hat{\text{y}}$_ (cf. Yin and Fan, 2001; Jiang and Smith, 2002). The findings from this study are unique as ρ ^2^_*y*$\hat{\text{y}}$_, *k* and *n* were also observed to contribute bias to *r*^2^_*x*$\hat{\text{y}}$_. This latter finding should not be surprising as it is logical these same factors influence sampling error and would therefore influence the bias of *R*^2^_*y*$\hat{\text{y}}$_ and *r*^2^_*x*$\hat{\text{y}}$_ (see discussion by Leach and Henson, 2007).

Researchers should be aware that when analyzing regression models with low to moderate amounts of explained variance in the presence of moderate to high amounts of multicollinearity, observed squared structure coefficients may underrepresent the predictive power of an independent variable in the population. Especially when the sample size/predictor ratio is 10 or less, the predictive power of an independent variable could be underrepresented by as much as 30%. Even with a more optimum sample size/predictor ratio of 10, our study revealed instances when the predictive power of an independent variable as measured by a uncorrected squared structure coefficient was underrepresented by as much as 10%. When considering Cohen's (1988) guidelines for the interpretation of variance accounted statistics, this amount of bias ranges between medium to large. Correcting observed structure coefficients using Pratt's formula is likely to yield less biased results with the exception of models with low amounts of explained variance.

The findings of the present study should be reviewed in light of the study's limitations. Our study considered a limited number of study conditions and the possibility exists that other study conditions might produce different results. An interesting scenario to study, for example, would be to consider conditions where validity coefficients and correlations among predictors were heterogeneous as in LeBreton et al. (2004). Another interesting scenario might be to conduct a study where specific values of ρ_*xy*_ were simulated independently of values of ρ ^2^_*y*$\hat{\text{y}}$_. In such a design, ρ_*xx*_ would therefore be chosen to yield desired levels of ρ ^2^_*y*$\hat{\text{y}}$_ given desired values of ρ_*xy*_. Future research might also examine other correction formulas than Pratt's. While Pratt's formula was chosen based on our review of the literature and has been touted as one of the best corrections for *R*^2^_*y*$\hat{\text{y}}$_ and *r^2^*, knowing how much better one formula does versus another could be informative. In the meantime, however, the realization that researchers might be able to report more accurate and stable estimates of ρ ^2^_*y*$\hat{\text{y}}$_ by computing *r*^2^_*x*$\hat{\text{y}}$_ from corrected *R*^2^_*y*$\hat{\text{y}}$_ and *r*^2^_*xy*_ with Pratt's formula should lead to the reporting of less biased results.

In a research world where multicollinearity is omnipresent, sample and effect sizes impact power, and the number of predictor variables affects regression results, we need to better understand how to minimize bias of *r*^2^_*x*$\hat{\text{y}}$_. Identifying the best correction formula will help in interpreting sample results that are more true to the population parameters. Even though the reporting of correction formulas has been recommended, many researchers are not adhering to those recommendations. In addition, when effect sizes have been reported, many authors do not report the correction formula used (Leach and Henson, 2007). Providing further evidence into the amount of bias exhibited and how to correct for this bias will help improve the validity of quantitative research.

The present study may also be beneficial for researchers as it serves as a foundation for multivariate analyses in the general linear model. For example, structure coefficients are utilized in many analyses within the general linear model. The results for multiple regressions, given similar conditions, should transfer to other analyses that produce structure coefficients. Utilizing decisions for one analyses based on another analyses is not new. For example, when conducting canonical correlation analyses, Sherry and Henson (2005) advocated cutoff values (i.e., 0.45) for noteworthy structure coefficients that typically have been used in exploratory factor analysis. Our study, therefore, could serve as a launching-off point to investigate corrections for structure coefficients for canonical correlation analyses, which subsumes all other analyses within the GLM (Knapp, 1978). One wonders, for example, to what degree decisions made to consider variables as noteworthy in canonical correlation based on fixed cutoff values of structure coefficients may have been influenced by bias. As noted by Thompson (1990), utilizing correction formulas in multivariate effect sizes can alert researchers for the need to conduct further analyses to resolve ambiguity when the noteworthiness of non-adjusted and adjusted effect sizes are not congruent.

Multiple regression has been a prevalent analyses in education research (Willson, 1980; Elmore and Woehlke, 1988; Kieffer et al., 2001; Zientek et al., 2008). Structure coefficients are essential to correct result interpretation in most cases (Thompson and Borrello, 1985; Courville and Thompson, 2001). Thus, reporting adjusted structure coefficients with the smallest amount of bias and then conducting follow-up analyses such as bootstrapping, cross-validation, or jackknife procedures can help researchers arrive at correct result interpretations.

### Conflict of interest statement

The authors declare that the research was conducted in the absence of any commercial or financial relationships that could be construed as a potential conflict of interest.

## References

[B1] Alhija F. N. Levy A. (2009). Effect size reporting practices in published articles. Educ. Psychol. Meas. 69, 245–265. 10.1177/0013164408315266

[B2] Bagozzi R. P. Fornell C. Larcker D. F. (1981). Canonical correlation analysis as a special case of a structural relations model. Multivariate Behav. Res. 16, 437–454. 10.1207/s15327906mbr1604_2 26812673

[B3] Claudy J. G. (1978). Multiple regression and validity estimation in one sample. Appl. Psychol. Meas. 2, 595–607. 10.1177/014662167800200414

[B4] Cohen J. (1968). Multiple regression as a general data-analytic system. Psychol. Bull. 70, 426–433. 10.1037/h0026714

[B5] Cohen J. (1988). Statistical Power Analysis for the Behavioral Sciences, 2nd Edn. Hillsdale, NJ: Erlbaum.

[B6] Courville T. Thompson B. (2001). Use of structure coefficients in published multiple regression articles: ß is not enough. Educ. Psychol. Meas. 61, 229–248. 10.1177/0013164401612006

[B7] Dunlap W. P. Landis R. S. (1998). Interpretations of multiple regression borrowed from factor analysis and canonical correlation. J. Gen. Psychol. 125, 397–407. 10.1080/00221309809595345

[B8] Elmore P. B. Woehlke P. L. (1988). Statistical methods employed in American Educational Research Journal, educational researcher, and review of educational research from 1978 to 1987. Educ. Res. 17, 19–20. 10.3102/0013189x017009019

[B9] Ezekiel M. (1929). The application of the theory of error to multiple and curvilinear correlation. J. Am. Stat. Assoc. 24, 99–104.

[B10] Fan X. (1997). Canonical correlation analysis and structural equation modeling: what do they have in common? Struct. Equ. Model. 4, 65–79. 10.1080/10705519709540060

[B11] Goodwin L. D. Goodwin W. L. (1985). Statistical techniques in *AERJ* articles, 1979–1983: the preparation of graduate students to read the educational research literature. Educ. Res. 14, 5–11.

[B12] Gorsuch R. L. (1983). Factor Analysis, 2nd Edn. Hillsdale, NJ: Erlbaum.

[B13] Graham J. M. (2008). The general linear model as structural equation modeling. J. Educ. Behav. Stat. 33, 485–506. 10.3102/1076998607306151

[B14] Graham J. M. Guthrie A. C. Thompson B. (2003). Consequences of not interpreting structure coefficients in published CFA research: a reminder. Struct. Equ. Model. 10, 142–153. 10.1207/S15328007SEM1001_7

[B16] Jiang Y. H. Smith P. L. (2002). Understanding and interpreting regression parameter estimates in given contexts: A Monte Carlo study of characteristics of regression and structural coefficients, effect size R squared and significance level of predictors. Paper presented at the annual meeting of the American Educational Research Association. New Orleans, LA. Retrieved from the ERIC database. (ED470301)

[B17] Johnson J. W. Lebreton J. M. (2004). History and use of relative importance indices in organizational research. Organ. Res. Methods 7, 238–257. 10.1177/1094428104266510

[B18] Kerlinger F. N. Pedhazur E. J. (1973). Multiple Regression in Behavioral Research. New York, NY: Holt, Rinehart and Winston.

[B19] Kieffer K. M. Reese R. J. Thompson B. (2001). Statistical techniques employed in *AERJ* and *JCP* articles from 1988 to 1997: a methodological review. J. Exp. Educ. 69, 280–309. 10.1080/00220970109599489

[B20] Kirk R. E. (1996). Practical significance: a concept whose time has come. Educ. Psychol. Meas. 56, 746–759. 10.1177/0013164496056005002

[B21] Knapp T. R. (1978). Canonical correlation analysis: a general parametric significance-testing system. Psychol. Bull. 85, 410–416. 10.1037/0033-2909.85.2.410

[B22] Kraha K. Turner H. Nimon K. Zientek L. R. Henson R. (2012). Tools to support Interpreting multiple regression in the face of multicollinearity. Front. Quant. Psychol. Meas. 3:44. 10.3389/fpsyg.2012.00044 22457655 PMC3303138

[B23] Leach L. A. Henson R. K. (2007). The use and impact of adjusted *R*^2^ effects in published regression research. Mult. Lin. Regression Viewpoints 33, 1–11.

[B24] LeBreton J. M. Ployhart R. E. Ladd R. T. (2004). A Monte Carlo comparison of relative importance methodologies. Organ. Res. Methods 7, 258–282. 10.1177/1094428104266017

[B25] Marszalek J. M. Barber C. Kohlkart J. Holmes C. B. (2011). Sample size in psychological research over the past 30 years. Percept. Mot. Skills 112, 331–348. 10.2466/03.11.PMS.112.2.331-348 21667745

[B27] Meredith W. (1964). Canonical correlations with fallible data. Psychometrika 29, 55–65. 10.1007/BF02289567

[B28] Nathans L. L. Oswald F. L. Nimon K. (2012). Interpreting multiple linear regression: a guidebook of variable importance. Pract. Assess. Res. Eval. 17, 1–19.

[B29] Olkin I. Pratt J. W. (1958). Unbiased estimation of certain correlation coefficients. Ann. Math. Stat. 29, 201–211. 10.1214/aoms/1177706717

[B30] Raju N. S. Bilgic R. Edwards J. E. Fleer P. F. (1999). Accuracy of population validity and cross-validity estimation: an empirical comparisons of formula-based, traditional empirical, and equal weights procedures. Appl. Psychol. Meas. 23, 99–115. 10.1177/01466219922031220

[B31] R Development Core Team. (2015). R: A Language and Environment for Statistical Computing. Vienna: R Foundation for Statistical Computing Available online at: http://www.R-project.org/

[B32] Sherry A. Henson R. K. (2005). Conducting and interpreting canonical correlation analysis in personality research: a user-friendly primer. J. Pers. Assess. 84, 37–48. 10.1207/s15327752jpa8401_09 15639766

[B33] Shieh G. (2008). Improved shrinkage estimation of squared multiple correlation coefficient and squared cross-validity coefficient. Organ. Res. Methods 11, 387–407. 10.1177/1094428106292901

[B34] Shieh G. (2010). Estimation of the simple correlation coefficient. Behav. Res. Methods 42, 906–917. 10.3758/BRM.42.4.906 21139158

[B35] Skidmore S. T. Thompson B. (2011). Choosing the best correction formula of the Pearson r^2^ effect size. J. Exp. Educ. 79, 257–278. 10.1080/00220973.2010.484437

[B36] Stevens J. P. (2002). Applied Multivariate Statistics for the Social Sciences, 4th Edn. Mahwah, NJ: Lawrence Erlbaum.

[B37] Taylor A. B. West S. G. Aiken L. S. (2006). Loss of power in logistic, ordinal logistic, and probit regression when an outcome variable is coarsely categorized. Educ. Psychol. Meas. 66, 228–239. 10.1177/0013164405278580

[B38] Thompson B. (1990). Finding a correction for the sampling error in multivariate measures of relationship: a Monte Carlo study. Educ. Psychol. Meas. 50, 15–31. 10.1177/0013164490501003

[B39] Thompson B. (2000). Ten commandments of structural equation modeling, in Reading and Understanding more Multivariate Statistics, eds Grimm L. Yarnold P. (Washington, DC: American Psychological Association), 261–284.

[B40] Thompson B. (2006). Foundations of Behavioral Statistics: An Insight-Based Approach. New York, NY: Guilford.

[B41] Thompson B. Borrello G. M. (1985). The importance of structure coefficients in regression research. Educ. Psychol. Meas. 45, 203–209.

[B42] Venables W. N. Ripley B. D. (2002). Modern Applied Statistics with S, 4th Edn. New York, NY: Springer.

[B43] Wang Z. Thompson B. (2007). Is the Pearson *r*^2^ biased, and if so, what is the best correction formula? J. Exp. Educ. 75, 109–125. 10.3200/JEXE.75.2.109-125

[B44] Willson V. L. (1980). Research techniques in *AERJ* articles: 1969 to 1978. Educ. Res. 9, 5–10.

[B45] Yin P. Fan X. (2001). Estimating *R*^2^ shrinkage in multiple regression: a comparison of different analytical methods. J. Exp. Educ. 69, 203–224. 10.1080/00220970109600656

[B46] Zientek L. R. Capraro R. M. Capraro M. M. (2008). Reporting practices in quantitative teacher education research: one look at the evidence cited in the AERA panel report. Educ. Res. 37, 208–216. 10.3102/0013189X0819762

[B47] Zientek L. R. Thompson B. (2009). Matrix summaries improve research reports: secondary analyses using published literature. Educ. Res. 38, 343–352. 10.3102/0013189X09339056

